# Genital and Extragenital Lichen Sclerosus et Atrophicus: A Case Series Written Using ChatGPT

**DOI:** 10.7759/cureus.38987

**Published:** 2023-05-13

**Authors:** Pratibha J P, Shruthi S Prasad, Naveen Manohar

**Affiliations:** 1 Department of Dermatology, St. John’s Medical College, Bangalore, IND; 2 Department of Dermatology, St. John's Medical College, Bangalore, IND; 3 Department of Dermatology, Belagavi Institute of Medical Sciences, Belagavi, IND

**Keywords:** depigmentation, artificial intelligence, chatgpt, autoimmune, extragenital, genital, lsea, lichen sclerosus et atrophicus

## Abstract

Background

Lichen sclerosus et atrophicus (LSEA) is a chronic inflammatory dermatosis of genital and extragenital sites with a prevalence ranging from 9% in prepubertal patients to 50% in postmenopausal patients. Chat generative pre-trained transformer (ChatGPT) is an artificial intelligence tool designed to assist humans based on supervised and reinforcement techniques. In this study, we aimed to evaluate the characteristics of patients with LSEA using ChatGPT.

Methods

In this retrospective study, we included all patients who presented to the outpatient dermatology department during 2017-2022 at a tertiary care teaching hospital in South India. Information regarding demographic data, characteristics of LSEA, comorbidities, and associated autoimmune disorders was gathered using a medical chart review. Following data analysis and drafting of the manuscript, the utility of ChatGPT-3 and ChatGPT-4 in finalizing the draft was assessed.

Results

Of 20 patients diagnosed with LSEA, 16 (80%) and four (20%) patients were females and males, respectively. Of them, 50% of female patients had attained menopause. While 65% of patients had genital LSEA, 30% of patients had extragenital LSEA only, and 5% of patients had both genital and extragenital LSEA. Furthermore, four (20%) patients were prepubertal children. Of four male patients, two (50%) were younger than 18 years of age, and one patient was diagnosed with balanitis xerotica obliterans. The commonest associated features in LSEA included joint involvement (30%), hypertension (25%), and anemia (15%). Rare concomitant disorders included psoriasis, asthma, and basal cell carcinoma over the nose.

Conclusions

LSEA may be confused with other various dermatoses, such as morphea, vitiligo, and lichen planus. A high index of suspicion is required, especially in children, to diagnose it early and intervene to prevent further complications. Its relationship with autoimmune disorders and comorbidities warrants further large-scale studies. ChatGPT was unreliable in the literature search due to the provision of non-existent citations. ChatGPT-4 was better than ChatGPT-3 since it provided few true publications. ChatGPT was used in this study to summarize the articles identified by the authors during the literature search and to correct grammatical errors in the final draft of the manuscript.

## Introduction

Lichen sclerosus et atrophicus (LSEA), also known as Csillag's disease [1], is a chronic inflammatory dermatosis that involves genital and extragenital sites. The prevalence of LSEA is 0.1%-0.3% of all patients referred to dermatology departments and ranges from 9% in prepubertal patients to 50% in postmenopausal patients [2]. Females are 10 times more commonly affected than males. Although anogenital involvement occurs in 85%-98% of cases, extragenital involvement is noted in 15%-20% of patients [3]. The manifestations of vulval LSEA include intractable itching, dyspareunia, and soreness as well as complications such as introital stenosis, atrophy of the labia majora, burying of the clitoris, and squamous cell carcinoma in very rare cases [1]. Kizer et al. reported that the incidence of LSEA in men was approximately 0.07% (n = 153,432); of them, 76% of their patients were 9-11 years of age [4]. Genital LSEA in males, also known as balanitis xerotica obliterans (BXO), can involve the foreskin, glans penis, frenulum, and meatus or urethra. The complications of BXO include phimosis and an association with penile squamous cell carcinoma [5].

LSEA may involve extragenital sites. The commonest extragenital sites include the trunk, neck, and upper limbs, and less common sites include the wrists, palms and soles, nipples, and face [6]. Isolated extragenital LSEA is noted in 2.5%-6% of all patients with LSEA [7]. The diagnosis of LSEA may be hampered by its presentation resembling other disorders, such as morphea [3], lichen planus [6], and vitiligo [8]. Baklouti et al. reported that the commonest extragenital sites of LSEA included the back (47%), breasts (41%), upper limbs (29%), lower limbs (29%), lower neck (17%), face (5%), and abdomen (5%) [7]. An association with autoimmune disorders such as vitiligo, thyroid disorder, and alopecia areata is seen in 74% of patients. Additionally, there is an association with infections such as human papillomavirus (HPV), hepatitis C, and *Borrelia burgdorferi* [1]. Due to such varied clinical presentations, a high index of suspicion is necessary to identify LSEA early and prevent its complications.

ChatGPT

Chat Generative Pre-Trained Transformer (ChatGPT; OpenAI, San Francisco, CA, USA) is an artificial intelligence (AI) chatbot that was released in November 2022 [9]. Flanagin et al. discussed how AI technologies are helping authors improve the quality of their manuscripts. These technologies include tools that assist with language, writing and grammar, references, statistical analysis, and reporting standards. Additionally, publishers and editors use AI-assisted tools for myriad purposes, including screening submissions for problems (e.g., plagiarism, image manipulation, and ethical issues), triaging submissions, validating references, editing, coding content, and facilitating post-publication discoverability. The authors of this study highlighted the responsibilities of authors; for example, if authors have used such tools, this must be transparently included in the methodology. However, these tools must be used with caution, and the responsibility lies with the authors [10].

In this study, we aimed to discuss the findings in genital and extragenital LSEA and their autoimmune associations. Additionally, we evaluated the role of ChatGPT in assimilating information and drafting the manuscript.

## Materials and methods

This retrospective observational study included all patients who were diagnosed with LSEA between January 2017 and December 2022 in the Department of Dermatology at St. John’s Medical College, Bangalore, India. Ethical clearance was obtained from the institutional ethics committee (approval number: IEC/1/480/2023). Informed consent was obtained from the patients or their guardians. This study was performed in accordance with the tenets of the Declaration of Helsinki 1964 and its subsequent revisions.

The medical records included complete history and examination findings of all the patients. The detailed history included questions to elicit symptoms and signs of autoimmune disorders, such as thyroid disorders (Hashimoto’s thyroiditis and Graves’ disease); autoimmune dermatological disorders, such as alopecia areata, vitiligo, systemic sclerosis, localized scleroderma, lupus erythematosus, pemphigus vulgaris, bullous pemphigoid, psoriasis, and dermatomyositis; Crohn’s disease and ulcerative colitis; and rheumatological disorders, such as rheumatoid arthritis (RA), Sjögren's syndrome, and ankylosing spondylitis. Following a complete physical examination, a 5-mm punch biopsy was performed for histopathological examination. The data collected included the age, sex, site of LSEA, symptoms, histopathological evidence, comorbidities, treatments prescribed, and any other pertinent medical information. Data of patients with incomplete information were excluded.

ChatGPT-3 was used to find relevant medical literature on the topic and convert articles into tables for easy readability. Additionally, the manuscript was drafted by the authors based on the information obtained and edited for language using ChatGPT-3. Furthermore, ChatGPT-4 was also used to collect newer data. The prompts used with ChatGPT-3 included the following: “How to write ChatGPT prompts for specific actions (e.g., grammar correction and tabulation)?”; “Summarize the following article into tables”; and “Edit the following text for grammar and style of an academic paper.” Additionally, the prompts used with ChatGPT-4 included the following: “Find newer articles on the following topic (e.g., ChatGPT in academic publishing)” and “Summarize the scholarly content of articles.” The content generated by the chatbot was reviewed by the authors. Non-existent information and misinformation were excluded. Additionally, the citations were identified by the authors based on a literature search. The authors take responsibility for the contents of this manuscript.

Statistical analysis

The data collected were recorded using a predefined proforma. Subsequently, they were entered into spreadsheets using Excel 365 (Microsoft Inc., Redmond, WA). The data are summarized and presented as mean ± standard deviation for continuous variables and number (percentages) for discrete variables.

## Results

Baseline data

In this study, we included 20 patients (females, n = 16; 80%; males, n = 4, 20%). The ages of the patients ranged from five years to 75 years with an overall mean age of 40.5 ± 24.5 years. The male patients were younger than the female patients (32.8 ± 29.2 vs. 42.4 ± 23.8 years, respectively). The gynecological status of female patients revealed that 50% of patients had attained menopause, while 25% of female patients were in the reproductive age group. Furthermore, 3/16 (18.8%) female patients were prepubertal children; in contrast, only one (25%) male patient was a prepubertal child of 11 years of age (Table 1).

**Table 1:** Baseline clinicodemographic data of patients with LSEA Data are presented as n (%) or mean ± standard deviation. LSEA: Lichen sclerosus et atrophicus.

Parameters	Females	Males	Overall
Number	16 (80%)	4 (20%)	20
Age, years	42.4 ± 23.8	32.8 ± 29.2	40.5 ± 24.5
Duration (months)	24.3 ± 35.5	4 ± 1.7	20.9 ± 33.1
Gynecological status			
Prepubertal	3 (18.8%)	-	-
Menstruating	4 (25%)	-	-
Menopausal	8 (50%)	-	-
Post-hysterectomy	1 (6.3%)	-	-
Site of LSEA			
Genital	11 (68.8%)	2 (50%)	13 (65%)
Extragenital	4 (25%)	2 (50%)	6 (30%)
Both	1 (6.3%)	0 (0%)	1 (5%)
Extragenital sites			
Upper limbs	2 (50%)	0 (0%)	2 (33.3%)
Trunk	4 (100%)	2 (100%)	6 (100%)
Lower limbs	2 (50%)	0 (0%)	2 (33.3%)
Biopsy-proven	16 (100%)	4 (100%)	20 (100%)

LSEA characteristics

**Figure 1:**
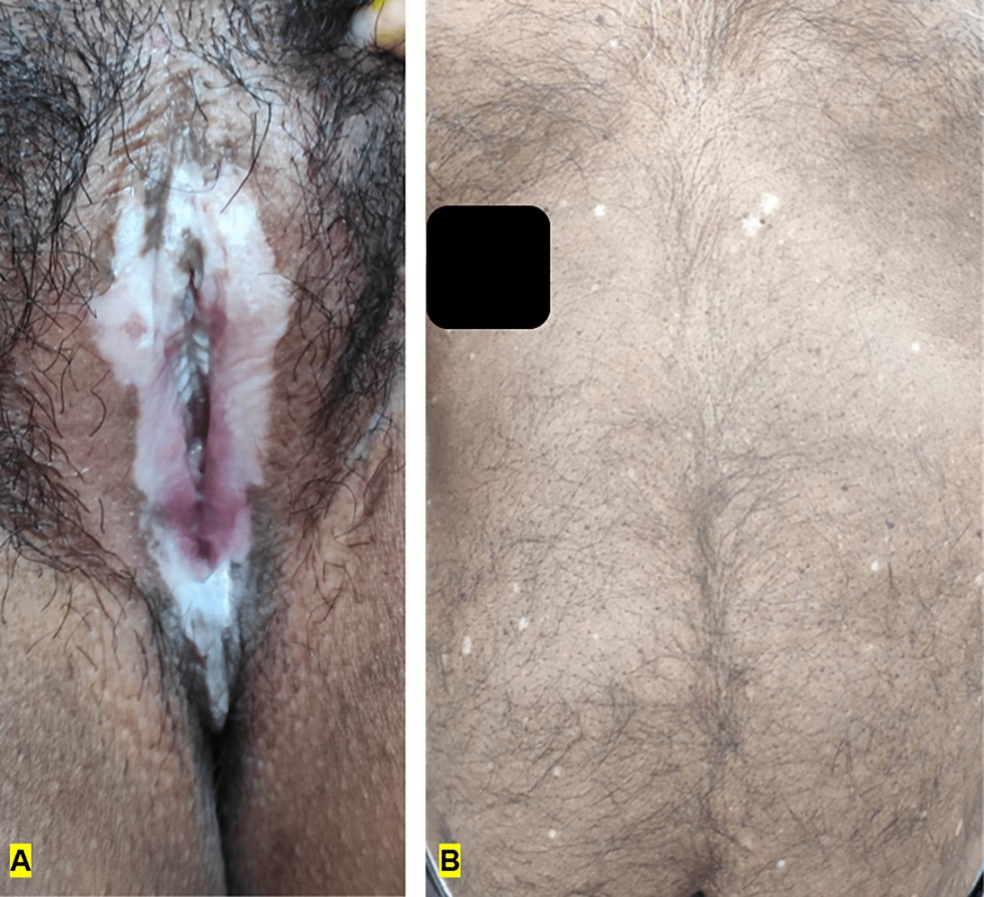
Genital and extragenital LSEA (A) Vulvar LSEA with warts in a 20-year-old female who was prescribed topical mometasone for two years. (B) Extragenital LSEA over the chest and abdomen in a 78-year-old man with diabetes mellitus and hypertension. LSEA: Lichen sclerosus et atrophicus.

The site of LSEA predominantly included the external genitalia (65%), while extragenital LSEA was noted in 30% of patients (Figure 1). Involvement of both genital and extragenital sites was noted in a single (5%) female patient. The commonest symptoms associated with LSEA included depigmentation (100%), followed by itching (45%) and burning sensation (31.3%).

**Figure 2:**
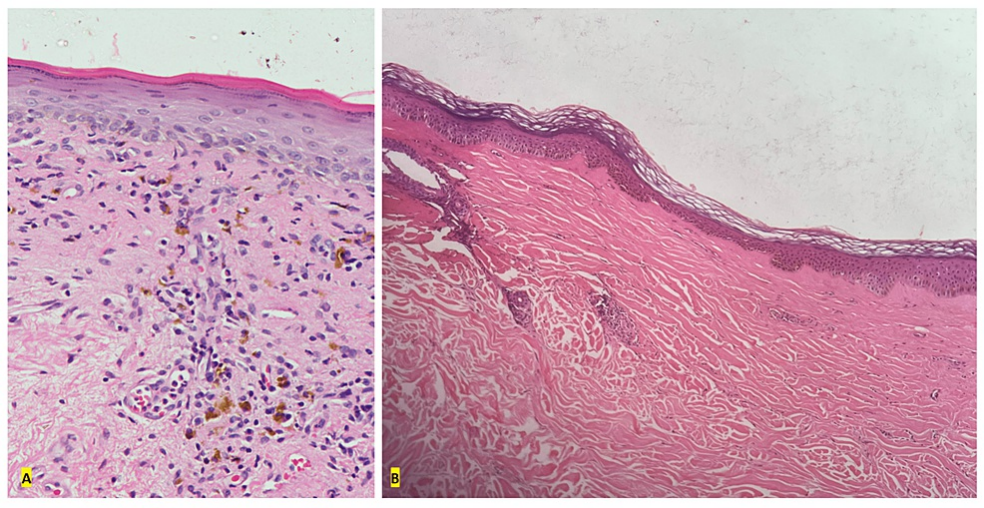
Histopathology in LSEA (A) Genital and (B) extragenital LSEA. The findings include dermal homogenization, flat rete ridges, epidermal atrophy, thinned granular layer, pigment incontinence, and dermal melanophages (H&E stain). LSEA: Lichen sclerosus et atrophicus.

Additionally, Koebnerization was noted in 35% of patients (Table 2). Histopathological evidence of LSEA was documented in all patients (100%) (Figure 2). The commonest treatment modalities used in the treatment of LSEA included potent topical corticosteroids (60%), antioxidants (45%), and topical calcineurin inhibitors (35%).

**Table 2:** Symptoms and comorbidities in patients with LSEA Data are presented as n (%). LSEA: Lichen sclerosus et atrophicus; DM: Diabetes mellitus; HTN: Hypertension; DLP: Dyslipidemia.

Parameters	Females	Males	Overall
Symptoms			
Burning	5 (31.3%)	0 (0%)	5 (25%)
Itching	8 (50%)	1 (25%)	9 (45%)
Depigmentation	16 (100%)	4 (100%)	5 (100%)
Urinary symptoms	0 (0%)	1 (25%)	1 (5%)
Koebnerization	6 (37.5%)	1 (25%)	7 (35%)
Comorbidities			
DM	1 (6.3%)	1 (25%)	2 (10%)
HTN	4 (25%)	1 (25%)	5 (25%)
Thyroid	2 (12.5%)	0 (0%)	2 (10%)
Joint involvement	6 (37.5%)	0 (0%)	6 (30%)
Anemia	3 (18.8%)	0 (0%)	3 (15%)
Axonal neuropathy	1 (6.3%)	0 (0%)	1 (5%)
DLP	3 (18.8%)	0 (0%)	3 (15%)

Interestingly, systemic immunosuppression was also prescribed, which included methotrexate (20%), systemic corticosteroids (10%), hydroxychloroquine sulfate (10%), and phototherapy (10%) (Table 3).

**Table 3:** Treatment prescribed in patients with LSEA Data are presented as n (%). LSEA: Lichen sclerosus et atrophicus; TCS: Topical corticosteroids; TCI: Topical calcineurin inhibitors; CS: Systemic corticosteroids; MTX: Methotrexate; HCQS: Hydroxychloroquine sulfate.

Parameters	Females	Males	Overall
TCS	10 (62.5%)	2 (50%)	12 (60%)
TCI	6 (37.5%)	1 (25%)	7 (35%)
Antioxidants	8 (50%)	1 (25%)	9 (45%)
CS	2 (12.5%)	0 (0%)	2 (10%)
MTX	4 (25%)	0 (0%)	4 (20%)
HCQS	2 (12.5%)	0 (0%)	2 (10%)
Phototherapy	2 (12.5%)	0 (0%)	2 (10%)

Disorders associated with LSEA

The commonest extracutaneous association with LSEA was noted with joint involvement (30%) in the form of arthralgia followed by hypertension (25%) and anemia (15%) (Table 2). However, uncommon comorbidities and disorders were noted as well. This included psoriasis and asthma in a postmenopausal 57-year-old female patient with genital LSEA. Similarly, the disorders in a 67-year-old female patient with extragenital LSEA included allergic rhinitis, dyslipidemia, hypertension, and hemiretinal vein occlusion. Another rare disorder included basal cell carcinoma on the nose in a 75-year-old male patient with extragenital LSEA.

## Discussion

In this study, we evaluated the clinical characteristics of patients with LSEA and evaluated the utility of ChatGPT-3 and ChatGPT-4 in drafting this paper. LSEA was more common in female patients than male patients; among various age groups, the highest prevalence was seen in postmenopausal women. The commonest site of involvement was the external genitalia (65%). Depigmentation was the commonest complaint (100%), and the commonest pharmacotherapy included topical corticosteroids (60%).

Virgili et al. reported that the commonest symptom in patients with vulvar LSEA (n = 225) was itching (90.2%) followed by burning (74.3%), dyspareunia (47.5%), and leukoderma (53.3%). Medical therapy alone was prescribed to 70% of their patients, and topical corticosteroids constituted the commonest therapy. Interestingly, they noted that 10% of their patients were initially prescribed topical antifungals, which suggests probable misdiagnosis presumably early in the course of LSEA without histopathological evaluations [11]. Early LSEA can mimic several disorders; Ganesan et al. highlighted the need for close monitoring in patients with isolated plaques with itching or hypopigmentation due to the possibility of LSEA transitioning to localized scleroderma or vice versa [3]. In extragenital LSEA, Baklouti et al. reported (n = 17) that the commonest site was the back (47%) [7]. In our study, we noted extragenital involvement in 30% of patients, and the chest was involved in all patients with extragenital LSEA. In all patients, 5-mm punch biopsies were performed for clinicopathological correlation (Figure 2).

The presentation of LSEA may resemble that of morphea [3], lichen planus [6], and vitiligo [8]. Patel et al. reported the case of a 39-year-old female who was clinically diagnosed with lichen planus, which was changed to LSEA based on histopathological features [6]. Attili et al. reported that in 74 patients with LSEA, 15 patients were clinically diagnosed with vitiligo initially. However, clinicopathological correlation resulted in a diagnosis of LSEA [8]. We did not encounter lesions resembling morphea or lichen planus; however, depigmented (100%) and asymptomatic vitiligo patches (25%) were noted, which were subsequently diagnosed as LSEA following histopathological evaluation.

Among infections, a probable association between LSEA and warts has been reported. Hadžavdić et al. reported two cases of adult male patients; one patient developed LSEA two years after undergoing cryotherapy for warts, while another patient who was diagnosed with LSEA developed warts eight weeks after starting topical 0.05% clobetasol propionate [12]. In our study, a 20-year-old female with LSEA was prescribed potent topical corticosteroids; six months later, she developed warts, which were resistant to conventional treatments (Figure 1). We suspect that impaired local immunity as a result of topical corticosteroids and immunosuppressants may explain the development of warts that are resistant to conventional treatment.

LSEA has been reported to be associated with several autoimmune conditions. Kreuter et al. reported that 15.4% of patients had autoimmune associations (n = 532), such as autoimmune thyroid diseases (7.9%), antithyroid antibodies (3.4%), and elevated antithyroid peroxidase (TPO) antibodies (6%) [13]. Thomas et al. reported autoimmune disorders in 21.5% of women with LSEA (n = 350), and the commonest disorder was alopecia areata [14]. In comparison, Harrington et al. reported an autoimmune association rate of 34% in 50 women with LSEA, and the commonest disorders included pernicious anemia and alopecia areata [15]. Another case study reported RA in a 21-year-old female African-American patient with LSEA [16]. Similar to these findings, autoimmune comorbidities in our patients included hypothyroidism (10%), elevated anti-TPO antibodies (5%), and RA (10%). One patient with genital LSEA was diagnosed with seropositive RA a year later; in contrast, another patient developed genital and extragenital LSEA 1.5 years following a diagnosis of seronegative RA. Furthermore, a 68-year-old female patient was diagnosed with genital LSEA followed by polymyalgia rheumatica 1.5 years later. Such associations can be explained by the presence of antibodies against human leukocyte antigen (HLA), which is alleged to act against the gastric parietal cells [15], intrinsic factors, thyroglobulin, and thyroid microsomes [14]. However, a higher prevalence of SLE, vitiligo, hypothyroidism, and autoimmune disorders is also noted with LSEA [13]. The precise mechanisms involved between LSEA and thyroid disorders remain unclear. However, both LSEA and autoimmune thyroid conditions involve abnormal T-lymphocyte activity. Autoreactive T-cells have been identified in vulvar tissues and peripheral blood in patients with LSEA, while T-cell autoreactivity against normal thyroid tissue is implicated in the pathogenesis of autoimmune thyroid disorders. Additionally, HLA alleles and molecular mimicry of microbial antigens may also play a role in the relationship between LSEA and thyroid disorders. Further studies are needed to completely understand such mechanisms [17].

Kiss et al. reported that the prevalence of BXO was 40% (n = 1178; age, 2-16 years) with the highest rate (76%) noted in those aged 9-11 years. Furthermore, secondary phimosis was noted in 93% of their patients [5]. In our study, of four male patients, two (50%) patients were aged 11 and 16 years, respectively. Furthermore, genital and only extragenital manifestations were noted in two (50%) patients each. Secondary phimosis was noted in one (25%) child, while urethral stricture was noted in one (25%) child who subsequently underwent post-meatal dilation. Notably, autoimmune associations were not noted in male patients. Kreuter et al. found that at least one autoimmune association was noted in 5.1% of their male patients with LSEA. The autoimmune thyroid disorders in these male patients included Hashimoto’s thyroiditis and Graves’ disease (3.8%) as well as RA and ulcerative colitis (0.7% each). Interestingly, the authors reported that women with LSEA were significantly more likely to have at least one autoimmune disease compared to men (odds ratio, 4.3; 95% confidence interval, 1.9-9.6; p < 0.0001) [13].

ChatGPT

We prompted ChatGTP-3 to draft an article on LSEA. Within a few seconds, the machine provided a complete article. The writing was logical and easy to read but included several repetitive sentences and generic content. Before moving any further, we repeated the prompt and asked for citations to published articles on the topic. However, a majority of the articles cited by ChatGPT were non-existent. Other authors have reported similar difficulties with non-existent citations by ChatGPT during the literature search. While Manohar et al. encountered this problem while drafting their case report and abandoned the tool [18], Haman and Školník found that 66% of the cited articles were non-existent, which is unacceptable for academic standards [19]. Clearly, the machine could communicate fluently but was severely flawed in finding reliable literature on the topic. Therefore, we decided to not use it to pursue the literature search any further.

**Figure 3:**
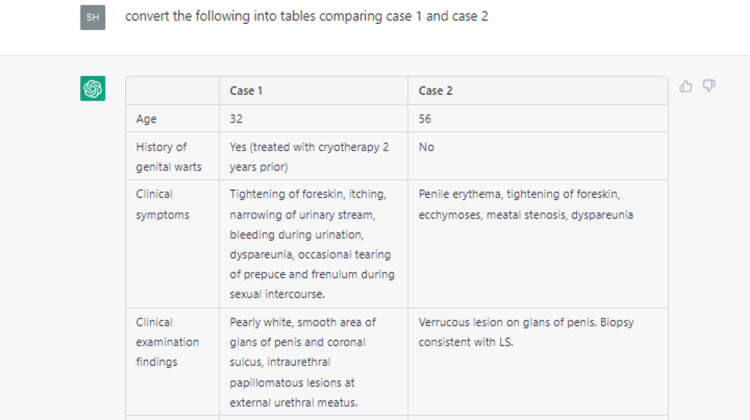
Prompting ChatGPT to summarize the text of a case report ChatGPT was able to summarize and tabulate the findings of two cases from the text. ChatGPT: Chat Generative Pre-trained Transformer.

One of the hurdles in publishing is the editing of the language in manuscripts. Although English is spoken in India, Indians are non-native speakers; consequently, grammatical and language errors can become a major barrier to publishing medical studies in international journals. Therefore, we can use tools, such as ChatGPT, to refine the language and highlight any language errors. Several apps and software tools are already available for this purpose. We did not evaluate the utility of ChatGPT in comparison to other tools. Instead, we chose to incorporate its help in summarizing articles that were chosen manually by us during a literature search on the topic (Figure 3). We began by identifying relevant articles on the topic online. Then, the text of the articles was extracted and provided to ChatGPT with a prompt to summarize the salient features in a tabular format for easy readability.

**Figure 4:**
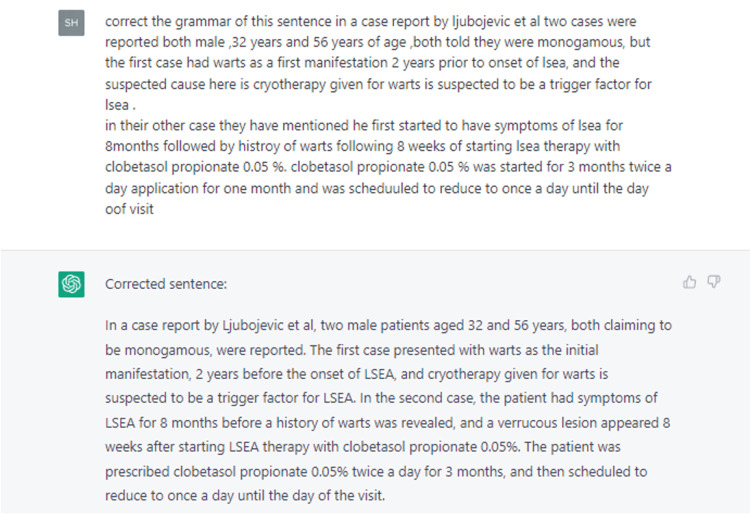
Using ChatGPT to edit text after drafting the manuscript. ChatGPT edited the paragraph for grammatical errors and punctuation. ChatGPT: Chat Generative Pre-trained Transformer.

Based on the points it had extracted, we drafted the manuscript and provided the draft to ChatGPT for language refinements. The tool was definitely helpful in identifying obvious errors and improving the tone of the paper for a more native presentation (Figure 4).

Presentation of research, theories, and arguments in a scientific style with adequate backing of reliable information is imperative in academic publishing. Formal language and tone along with a clear structure and a focus on evidence and analyses distinguishes academic publications. Originality in academic writing allows for advancements in knowledge while promoting critical thinking and creativity. Good-quality academic writing should contribute to the existing body of knowledge while attempting to present new insights, perspectives, or arguments. Originality also helps in keeping plagiarism in check and upholds the ethical standards of academic writing [20]. Liebrenz et al. contemplated the ethical implications of using ChatGPT in academic publishing. They discussed if the use of ChatGPT should be considered plagiarism since authors will be using content not written by themselves. Additionally, the question of copyright and intellectual property over the content presented by AI remains to be discussed further. Once platforms, such as ChatGPT, are monetized, the international inequities in scholarly publishing may worsen rather than improve [9].

ChatGPT-3 is limited by the information that was available till 2021, which makes it unsuitable for a literature search on the latest advancements in current topics. ChatGPT-4 is the newer version that is accessible via Microsoft’s Bing Chat (Microsoft Inc., Redmond, WA) and includes updated information that is continuously revised. ChatGPT-4 is only available as a paid service, while Bing Chat includes a free variant. We used Bing Chat to search for articles on the topic with mixed results. Some of the suggested articles were real, which is an improvement from our previous experience. However, it also sent us on a wild goose chase across websites on housing, banking, and other topics that were irrelevant to our work. Therefore, we rejected its use for literature search. Instead, we used it to address any grammatical and language errors in the manuscript; it was able to refine our manuscript for grammatical errors while also suggesting more citations, some of which were non-existent. Interestingly, ChatGPT-4 was able to summarize articles without providing any specific answers to our questions regarding topics contained within the article. Consequently, we used its assistance only to correct the grammar of our finalized draft of this manuscript but did not use its suggested citations or literature search results.

## Conclusions

In our retrospective cohort of patients with LSEA, 80% of patients were women, and 50% of women had attained menopause. Extragenital LSEA was seen in 30% of patients with LSEA, and the commonest extracutaneous manifestation included joint involvement (30%) followed by hypertension (25%). ChatGPT is still evolving, and it holds promise as an excellent assistant tool in academic publication with its newer iterations. However, several gray areas require further discussions, such as authorship, ethics, and legality. ChatGPT may appear to be a genie that can grant wishes based on our prompts, but as with stories about genies, it is our responsibility to ensure that we are not tricked into misinformation and perilous situations due to irrelevant information.
